# Coacervation in systems chemistry

**DOI:** 10.1038/s42004-024-01358-1

**Published:** 2024-11-22

**Authors:** Lorraine Leon, Guillermo Monreal Santiago, Evan Spruijt

**Affiliations:** 1 Materials Science and Engineering, University of Central Florida, Orlando, FL USA; 2 UMR7140 – Chimie de la Matière Complexe, Université de Strasbourg, CNRS, Strasbourg, France; 3 Institute for Molecules and Materials, Radboud University, Nijmegen, the Netherlands

**Keywords:** Origin of life, Self-assembly, Soft materials

## Abstract

*Communications Chemistry* is delighted to introduce a Collection of articles that combine coacervation with systems chemistry. Here, the Guest Editors of the Collection introduce the importance of research at the interface between these two topics and highlight the current challenges in this area.

## Coacervate compartments as a tool toward complexity

Some of the most fascinating properties in chemistry cannot be assigned to individual molecules or reactions, but only arise from networks of interconnected molecules that react and interact with each other. Oscillations, self-organization, pattern formation, and self-replication are examples of these emergent properties. Studying reaction networks is particularly important in connection to biology, as living organisms can only be understood holistically. Therefore, any chemical designs that aim to either interact with life or recreate life need to be done from a systems perspective. The field that studies these relationships is called systems chemistry.

A key step towards achieving life-like complexity is compartmentalization. The spatial segregation of different parts of a chemical system into compartments, which only partially communicate with each other, has been shown to have surprising effects in reaction networks. Examples of these effects are a change in kinetics upon compartmentalization, directing an out-of-equilibrium network of reactions towards different products^1^, or a different ecological regime of self-replicators, in which negative mutations (e.g. parasitism) can be overcome by the introduction of transient compartments^2^.

In particular, coacervates have been shown to be excellent compartments for chemical reaction networks, due to their properties such as their facile preparation, which can occur by mixing oppositely charged polymers for complex coacervates, or changing solution conditions (pH, temperature, etc.) for single component (simple) coacervates. Similarly, properties of the coacervate phase, such as viscosity and permeability, can be tuned easily through solution conditions, including facilitating the dissolution of the coacervate itself^3^, which could be controlled via chemical reactions. Coacervates have an ultra-low interfacial tension with water, making them good at the encapsulation of molecules, which can be engineered to be selective^4^. However, this low interfacial tension often causes their coalescence, leading to significant dispersity in the size of coacervate droplets. Coacervates have a lot of similarities with liquid-liquid phase separations that occur within cells, often called membrane-less organelles or biomolecular condensates. Indeed, many efforts, including those in this Collection, have involved using coacervates as mimics of biomolecular condensates^5^. Beyond intracellular structures, the compartmentalization provided by the coacervate phase has also been used in the construction of synthetic cells and protocells, where the concentrated coacervate phase is meant to mimic the crowded cell interior.

Coacervates thus introduce an intriguing new level of complexity to chemical reaction networks, with significant potential for the construction of synthetic systems with emergent life-like properties, such as catalysis, communication, growth, and division. Interestingly, some of the key challenges involved in studying the role of coacervates on chemical reaction networks and designing tailored compartments with specific chemical and physical properties stem from the same interdependencies that define systems chemistry^6^.

## Understanding coacervate properties and how they can change

Because of their phase-separated nature, many physicochemical properties of coacervates are intimately related and ultimately determined by the underlying intermolecular interactions: changing the molecular structure of the coacervate components can alter the phase boundary of the coacervate, but also its surface tension, density, and material properties. This poses an important challenge for the design of coacervates with specific reversibility and material properties, for example for synthetic cells. In this Collection, Dong Soo Hwang and co-workers systematically investigate the influence of backbone chemistry and the chemical nature of the charged groups of polyelectrolytes on the formation and properties of complex coacervates^7^. Related, Ali Miserez and co-workers show that the phase boundary and viscoelastic properties of peptide-based coacervates can be tuned by single amino acid mutations and variations in the type of salt ions that are added^8^, while Deshpande and co-workers show that such amino acid mutations can be exploited to create coacervate organelles inside a synthetic cell compartment reversibly in response to pH, osmolarity, or temperature changes^3^. DNA is another versatile molecule that can be used to create coacervates with highly tunable properties. Walther and co-workers engineer coacervates made of DNA with tunable stability, material properties, and interaction with other coacervates or membranes by adding DNA or RNA with specific sequences and lengths^9^. Gül Zerze and co-workers further highlight the use of computer simulations to understand the molecular changes inside a coacervate when the surrounding environment is changed, for example, to stabilize the coacervate droplets against coalescence^10^.

## Controlled molecular uptake and release

Many applications of coacervates require the selective uptake and release of molecules, for example as substrates for reactions taking place inside, or for the release of signaling cues or molecules with a therapeutic function. Designing coacervate systems that allow for control over this uptake or release, with specificity at the molecular level, is a key challenge for many of the applications for coacervates. Luc Brunsveld, Jan van Hest, and co-workers describe how the recruitment and release of specific proteins in coacervates could be controlled by the hub protein 14-3-3, featuring varying protein-protein interaction affinities^4^. Lampel and co-workers show that enzymatic reaction rates inside coacervates can be tuned by varying the coacervate composition^5^. Jiahua Wang, Yuehua Li, and co-workers achieve chemical and enzymatic control over the release of proteinous drugs from coacervates through redox reactions^11^. Another method to control the uptake or release by coacervates is to coat them with a membrane with tunable fluidity and thickness. Yanglimin Jin and Yan Qiao present a method to create dextran-based membranes around complex coacervates and investigate their fluidity and permeability^12^.

## From directing reactions to functional diversity

As control over the physicochemical properties of coacervates and the uptake and release of molecules gets improved, coacervates become a versatile tool to modulate chemical reactions and reaction networks selectively. This brings applications in which coacervates can be used to either interact with living systems or recreate life-like functions within reach. Several papers in this Collection illustrate the potential for coacervates to modulate chemical reactions, assembly processes, communication, and the construction of higher-order hierarchical structures. Starting from a fundamental understanding of reactions in coacervates, Siewert Jan Marrink and co-workers show how reactive molecular dynamics simulations can be used to capture and better understand chemical reactions inside coacervates^13^. Shashi Thutupalli and co-workers then demonstrate that coacervates can not only alter the rates of self- and cross-catalytic reactions but also endow robustness to the collective autocatalytic network^14^. The unique microenvironment inside coacervates can direct covalent reactions but also modulate noncovalent interactions and self-assembly. Sebastián Díaz and co-workers show that coacervates can improve the kinetics and detection limit of fluorescence-based DNA biosensors that rely on DNA hybridization^15^. Javier Montenegro and co-workers review how coacervates can influence the formation of supramolecular fibrils^16^. The coacervates themselves can also be used to build higher-order structures for use in synthetic biology or biomedical applications. Jianwei Li and co-workers review how coacervates could be stabilized and self-assembled into such higher-order structures^17^. Finally, when coacervates are stabilized and release from coacervates can be controlled, they could be used to establish communication with living cells, and Hongjing Dou and co-workers review the three modes of communication in consortia of synthetic and living cells^18^.

## Outlook

Coacervates hold great potential to create and control reactive chemical systems with emergent properties, as a framework for understanding the chemical principles of some of the hallmarks of life, but also to build synthetic cell-like compartments. At the same time, designing and studying coacervates with increasing complexity and functional diversity brings fundamental challenges, for which a systems chemistry perspective is indispensable. Coacervates and systems chemistry are thus inherently connected.

This Collection includes some state-of-the-art research, directed at both tackling these fundamental challenges and at the development of complex systems involving coacervates. We hope these Reviews and Articles will inspire further exciting research and encourage interactions between scientists from the various contributing fields.
